# Supplementary material for: COZOID: contact zone identifier for visual analysis of protein-protein interactions
Source: BMC Bioinformatics. 2018 Apr 6;19:125. doi: 10.1186/s12859-018-2113-6 (PMC5889581; doi:10.1186/s12859-018-2113-6)
Supplement: Supplementary file 2 — Software build. Executable binary file of the software tool. (ZIP 118,784 kb) [file 12859_2018_2113_MOESM2_ESM.zip › cozoid/java/docs/beansbinding-1.2.1-doc.zip!/org/jdesktop/beansbinding/class-use/ObjectProperty.html]

Uses of Class org.jdesktop.beansbinding.ObjectProperty (Beans Binding)


---


|  |  |  |
| --- | --- | --- |
| **Overview** \| **Package** \| **Class** \| **Use** \| **Tree** \| **Deprecated** \| **Index** \| **Help** | |  |
| PREV   NEXT | **FRAMES**    **NO FRAMES**     **All Classes** |  |


---


## **Uses of Class org.jdesktop.beansbinding.ObjectProperty**

| Packages that use ObjectProperty | |
| --- | --- |
| **org.jdesktop.beansbinding** | Provides support for defining properties and creating bindings between sets of two properties. |

Uses of ObjectProperty in org.jdesktop.beansbinding

Methods in org.jdesktop.beansbinding that return ObjectProperty

`static

<S> ObjectProperty<S>`

`ObjectProperty.create()`
  
          Creates an instance of `ObjectProperty`.

---


|  |  |  |
| --- | --- | --- |
| **Overview** \| **Package** \| **Class** \| **Use** \| **Tree** \| **Deprecated** \| **Index** \| **Help** | |  |
| PREV   NEXT | **FRAMES**    **NO FRAMES**     **All Classes** |  |


---
